# Montelukast Inhibits Lung Cancer Cell Migration by Suppressing Cysteinyl Leukotriene Receptor 1 Expression *In vitro*

**DOI:** 10.2174/1389201024666221207143513

**Published:** 2023-05-05

**Authors:** Yisheng Chen, Jinye Zhang, Shuo Wei

**Affiliations:** 1 Center for Experimental Research in Clinical Medicine, Shengli Clinical Medical College of Fujian Medical University, Fujian Provincial Hospital, Fuzhou, China;; 2 Department of Infectious Disease, Shengli Clinical Medical College of Fujian Medical University, Fujian Provincial Hospital, Fuzhou, China

**Keywords:** Lung cancer, montelukast, CRISPR/Cas9, CysLT1, migration, drug repositioning

## Abstract

**Background:**

Lung cancer is a major threat to public health and remains difficult to treat. Repositioning of existing drugs has emerged as a therapeutic strategy in lung cancer. Clinically, low-dose montelukast has been used to treat asthma.

**Objective:**

We evaluated the potential of using montelukast to treat lung cancer.

**Methods:**

Migration was detected using wound-healing and Transwell assays, the expression of CysLT1 using western blotting, and subcellular localization of CysLT1 using immunofluorescence. CRISPR/Cas9 technology was used to further investigate the function of CysLT1.

**Results:**

Subcellular localization staining showed that the CysLT1 distribution varied in murine and human lung cancer cell lines. Furthermore, montelukast suppressed CysLT1 expression in lung cancer cells. The treated cells also showed weaker migration ability compared with control cells. Knockout of *CysLT1* using CRISPR/Cas9 editing in A549 cells further impaired the cell migration ability.

**Conclusion:**

Montelukast inhibits the migration of lung cancer cells by suppressing CysLT1 expression, demonstrating the potential of using CysLT1 as a therapeutic target in lung cancer.

## 1 INTRODUCTION

The morbidity and mortality rates associated with lung cancer have increased worldwide because of pollution and changes in living habits, creating major challenges for public health and economic development. In China, 787,000 individuals were diagnosed with cancer, and lung cancer incidence was approximately 20% in 2015 [1]. Lung cancer has a mortality rate of 30%, representing a threat to global health. Brain metastasis of tumor cells is common in malignancies [2-4]. Approximately 50% of advanced non-small cell lung cancer (NSCLC) patients develop brain metastases [5], which is associated with a poor prognosis. Numerous studies of lung cancer have been conducted to improve precision therapies and have led to the development of molecular-targeted agents [6]. For example, the conventional drug osimertinib inhibits epidermal growth factor receptor tyrosine kinase and is used to prevent brain metastases in NSCLC [7]. However, drug resistance is a major limitation in targeted therapy, leading to a substantial proportion of non-responsive patients. Although novel, effective drugs are urgently needed, the high costs of drug development and the long developmental cycle have prevented major advances in therapeutic approaches. Repurposing existing drugs may be an alternative approach to improving anticancer therapies. Chronic inflammation is associated with tumor occurrence and development [8] and contributes to tumor growth by promoting cellular proliferation and migration, reducing apoptosis, and triggering DNA damage [9-12]. Cysteinyl leukotriene, an important mediator of persistent inflammation [13], is derived from arachidonic acid, a metabolic product of the 5-lipoxygenase pathway. Among the five subtypes of cysteinyl leukotrienes, three (leukotriene C4, leukotriene D4 [LTD^4^], and leukotriene E4) are secreted from cells via the cell membrane [14]. LTD4 promotes lung cancer cell survival and migration [15]. Two G-protein-coupled receptors specific for cysteinyl leukotriene, cysteinyl leukotriene receptor 1 (CysLT1) and cysteinyl leukotriene receptor 2, have been identified [16]. The cysteinyl leukotriene receptor antagonist montelukast is commonly used to treat asthma in clinical settings [17]. Montelukast also inhibits lung cancer and glioblastoma metastasis and induces apoptosis in lung cancer cells [18, 19]. In the lungs, montelukast antagonizes CysLT1 by inhibiting the binding of LTD4 to CysLT1, thus disrupting an interaction closely associated with lung cancer development and progression. Additionally, cPLA2-5-LOX-CysLT mediates cryptococcus entry into the central nervous system *via* CysLT1 [20]. Because this process is similar to the brain metastasis of lung cancer cells, we hypothesized that CysLT1 is involved in lung cancer. Accordingly, this study examined whether montelukast can be repositioned as a preventive treatment for lung cancer cell metastasis. We evaluated the mechanisms of action by which montelukast inhibits lung cancer cell migration and examined CysLT1 functions using CRISPR/Cas9 technology.

## 2 MATERIALS AND METHODS

### 2.1 Cell Culture

The human lung carcinoma cell line A549 and mouse lung carcinoma cell line LLC were purchased from the Cell Line Bank, Chinese Academy of Sciences (Shanghai, China). A549 cells were cultured in Ham’s-F12K (Shanghai Basal Media Technologies, Shanghai, China), and LLC cells were cultured in Dulbecco’s modified Eagle’s medium (Shanghai Basal Media Technologies) supplemented with 10% fetal bovine serum (FBS; Gibco, Grand Island, NY, USA) and 100 μg/mL penicillin/streptomycin (Gibco). Both lung carcinoma cell lines were cultivated at 37°C in a 5% CO_2_ incubator (Thermo Fisher Scientific, Waltham, MA, USA).

### 2.2 *CysLT1* Knockout

The CRISPR/Cas9 system was used to construct a *CysLT1*-knockout A549 cell line. The target human *CysLT1* single-guide RNA (sgRNA) was designed using the CRISPR design website of the Feng Zhang Lab (http://crispr.mit.edu/). The primers used for polymerase chain reaction (PCR) were designed using Primer-BLAST (https://www.ncbi.nlm.nih.gov/tools/primer-blast/). After PCR, knockout of the target sequences was evaluated using agarose gel electrophoresis. All oligos (*CysLT1*-knockout target sites) and primers were synthesized by Sangon Biotech Co., Ltd. (Shanghai, China). Complementary oligonucleotides of the sgRNA were annealed and cloned into the pSpCas9 (BB)-2A-puro vector (PX459; Addgene, Watertown, MA, USA). Plasmids containing the sgRNA were transferred into A549 cells using Lipofectamine 3000 (Thermo Fisher Scientific) according to the manufacturer’s protocol and treated with 2 μg/mL puromycin (Solarbio, Beijing, China) after 48 h. The cells were screened with puromycin, and single A549 cells were isolated and inoculated into 96-well plates. PCR and Sanger sequencing (Sangon Biotech) were performed 2 weeks later.

### 2.3 DNA Extraction and PCR

DNA was extracted from the cells using DNAzol (Thermo Fisher Scientific) according to the manufacturer’s recommendations. PCR primers were designed for *CysLT1* (forward: 5′-CAGGAGGGCTGTTTCACCTA-3′, reverse: 5′-TGATTGTCTTGTGGGGGCTC-3′), and an 844-bp fragment of *CysLT1* was amplified. The PCR products were visualized *via* agarose gel electrophoresis.

### 2.4 Western Blot Analysis

Total protein lysates were collected using RIPA buffer (Beyotime Biotechnology, Jiangsu, China). Thermally denatured proteins were loaded onto 10% polyacrylamide gels, separated by sodium dodecyl sulfate-polyacrylamide gel electrophoresis at a constant voltage, and transferred to polyvinylidene difluoride membranes. The membranes were blocked with anti-CysLTR1 polyclonal antibodies (EpiGentek, Farmingdale, NY, USA) or anti-β-actin antibodies (ProteinTech, Rosemont, IL, USA) overnight at 4°C and then incubated with appropriate secondary antibodies for 1 h at 25°C. The membranes were washed with Tris-buffered saline containing Tween 20 and visualized using an enhanced chemiluminescence kit (Thermo Fisher Scientific).

### 2.5 Wound-healing Assay

A549 or A549^−/−^ cells (10^6^ cells/mL) were seeded into 96-well plates and incubated for 24 h in a medium supplemented with 10% FBS until reaching approximately 100% confluence. Similar straight scratches were created in the center of each well, after which the cells were serum-starved for 2 h. The culture medium was replaced with a medium containing 2% FBS and various concentrations of montelukast (0, 25, or 50 μM). The cells were incubated for 48 h, and a high-content imaging system (Molecular Devices, Sunnyvale, CA, USA) was used to capture images continuously for 48 h (magnification: 100×). The areas between the wound edges were measured and analyzed using ImageJ software (V1.8.0; National Institutes of Health, Bethesda, MD, USA).

### 2.6 Transwell Migration Assay

LLC cell migration was examined using transwell chambers with 8 μm pores. LLC cells (10^4^ cells/well) suspended in serum-free medium were added to the upper transwell chambers (Thermo Fisher Scientific). Medium supplemented with 10% FBS was added to the bottom wells to stimulate migration, and the cells were incubated for 24 h. Residual cells on the upper surface of the polycarbonate membrane were removed using cotton swabs. Migrated cells on the lower surface of the chambers were fixed with 4% paraformaldehyde solution (Solarbio) for 30 min and stained with 0.5% crystal violet (Beyotime Biotechnology) for 10 min at 25°C. Images were captured using a light microscope (Leica, Wetzlar, Germany).

### 2.7 Cellular Immunofluorescence Assay

The cells were attached to glass slides, fixed with 4% paraformaldehyde, incubated with anti-CysLTR1 polyclonal antibodies (EpiGentek), and incubated with Alexa Fluor 488-conjugated secondary antibodies (Beyotime Biotechnology). The slides were counterstained with 4′,6-diamidino-2-phenylindole (5 mg/mL; Beyotime Biotechnology), and images were captured using a fluorescence microscope (Leica).

### 2.8 Statistical Analysis

All data are presented as the mean ± standard deviation. Western blotting results were evaluated using *t-*tests. One-way analysis of variance was used to analyze the transwell assay results. Differences with *p-*values less than 0.05 were considered as statistically significant.

## 3 RESULTS

### 3.1 Montelukast Inhibits A549 Cell Migration

**Fig. (1):**
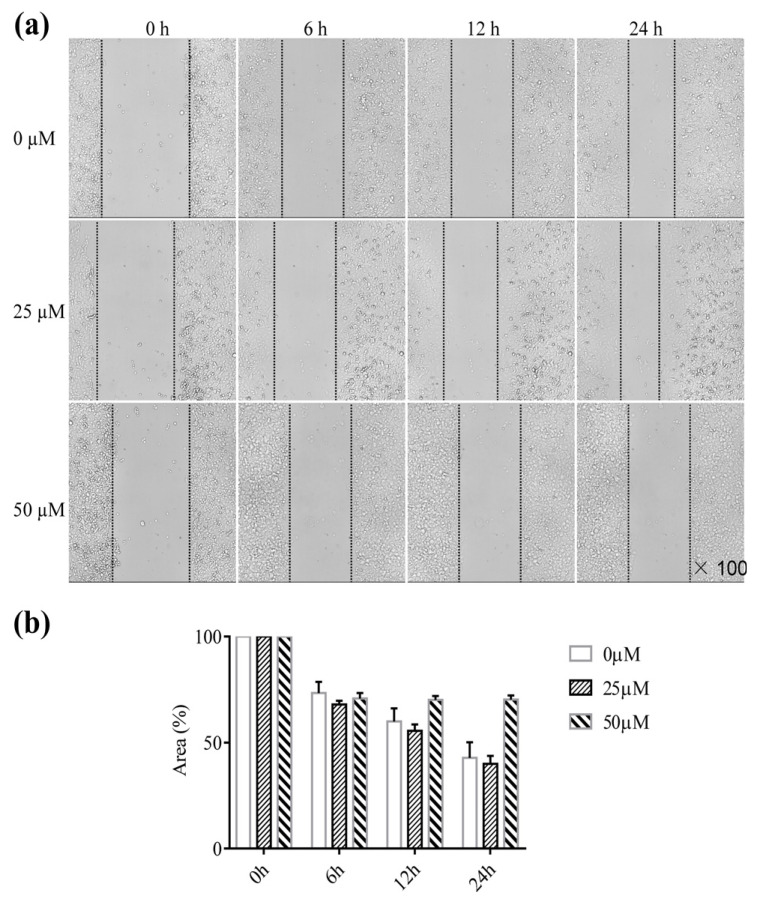
Montelukast inhibits the migration of A549 cells. (**a**) Migration ability was detected over 24 h in the presence or absence of the indicated concentrations of montelukast using wound-healing assays. (**b**) Ratio of the change in wound area calculated using high-content imaging. 0 µM: untreated A549 cells; 25 µM: A549 cells treated with 25 µM montelukast; 50 µM: A549 cells treated with 50 µM montelukast. Data are presented as means±SDs (n = 4). *p < 0.05.

**Fig. (2):**
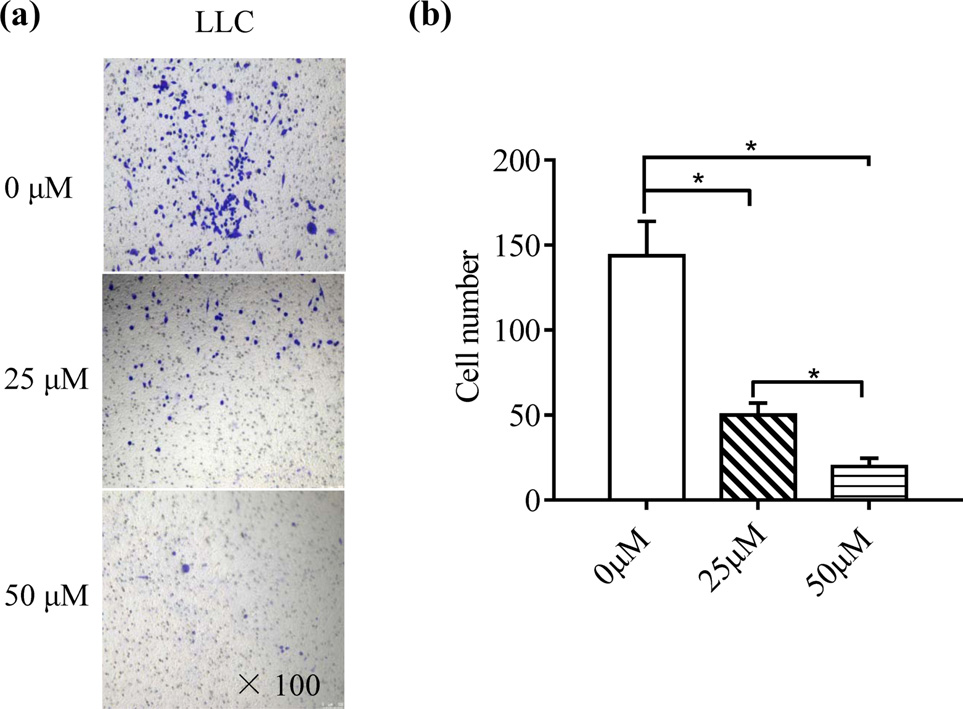
Transwell migration assays of LLC cells. (**a**) Representative images of transwell assay on LLC cells (magnification: 100×). (**b**) Cell migration to the lower compartment within 24 h in the presence or absence of the indicated treatments. 0 µM: untreated LLC cells; 25 µM: LLC cells treated with 25 µM montelukast; 50 µM: LLC cells treated with 50 µM montelukast. Data are presented as the mean±SD (n = 5). *p < 0.05.

To investigate whether montelukast inhibits cell migration, A549 cells were treated with different montelukast concentrations. Scratch assays were performed, and changes in the wound area were measured every 6 h. There was no difference between the 25 µM montelukast and control groups; however, treatment with 50 µM montelukast markedly inhibited cell migration (Fig. **1**). These findings suggest high concentrations of montelukast can regulate lung cancer cell migration. To confirm the effects of montelukast on different lung cancer cells, the migratory capacity of semi-adherent LLC cells was assessed in transwell migration assays. The results showed that montelukast treatment reduced the migration of LLC cells in a concentration-dependent manner (Fig. **2**).

### 3.2 Subcellular Localization of CysLT1 in Lung Cancer Cells

**Fig. (3):**
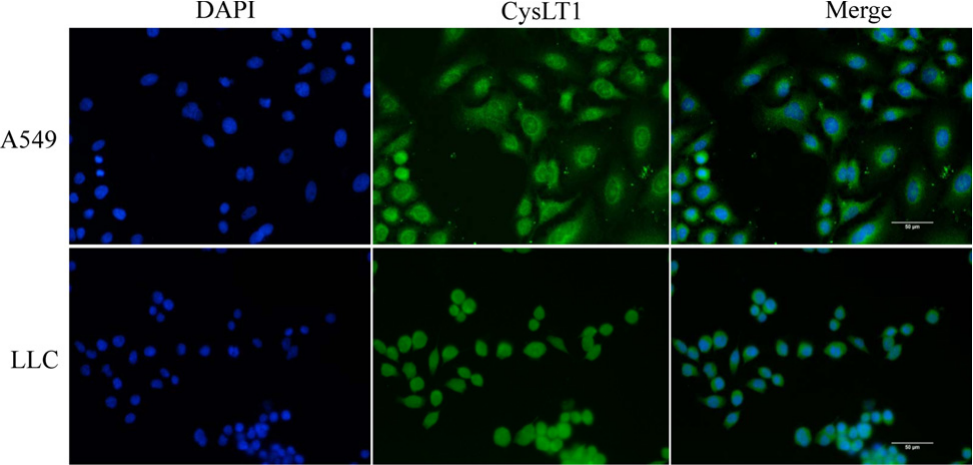
Spatial localization of CysLT1 in A549 and LLC cells. Blue fluorescence represents nuclei stained with 4′, 6-diamidino-2-phenylindole, and CysLT1 is labeled with green fluorescence. Scale bar = 50 μm.

To evaluate CysLT1 localization in different lung cancer cells, immunofluorescence assays were performed. The results showed that the distribution of CysLT1 in lung cancer cells varied between the cell lines (Fig. **3**). In human A549 cells, CysLT1 was detected around the nucleus and sparsely distributed on the cell membrane, whereas in murine LLC cells, CysLT1 was observed in the cell membrane.

### 3.3 Montelukast Suppresses CysLT1 Expression in A549 Cells

**Fig. (4):**
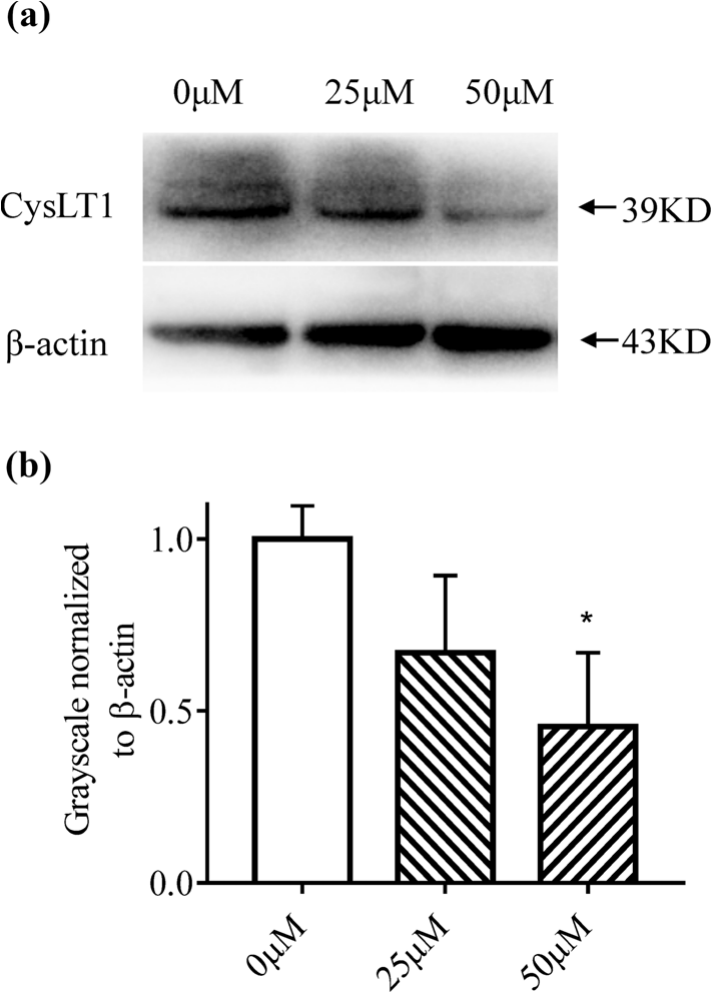
Effects of montelukast on CysLT1 expression in A549 cells. (**a**) A549 cells were treated with different concentrations of montelukast, and CysLT1 protein levels were evaluated using western blotting. (**b**) Gray value of each protein band was determined using ImageJ software, and data were normalized to β-actin expression, with the value of the 0 μM group set at 1.0 µM: untreated A549 cells; 25 µM: A549 cells treated with 25 µM montelukast; 50 µM: A549 cells treated with 50 µM montelukast. Data are presented as the mean±SD (n = 3). *p < 0.05.

To investigate the effects of montelukast on CysLT1 expression, CysLT1 levels were detected using western blotting. CysLT1 expression was clearly suppressed following treatment with 50 μM montelukast (*p* < 0.05), whereas 25 μM montelukast had no significant effect on CysLT1 expression (*p* > 0.05) (Fig. **4**). The results confirm that CysLT1 expression was inhibited by high-concentration montelukast.

### 3.4 *CysLT1* Knockout in A549 Cells

**Fig. (5):**
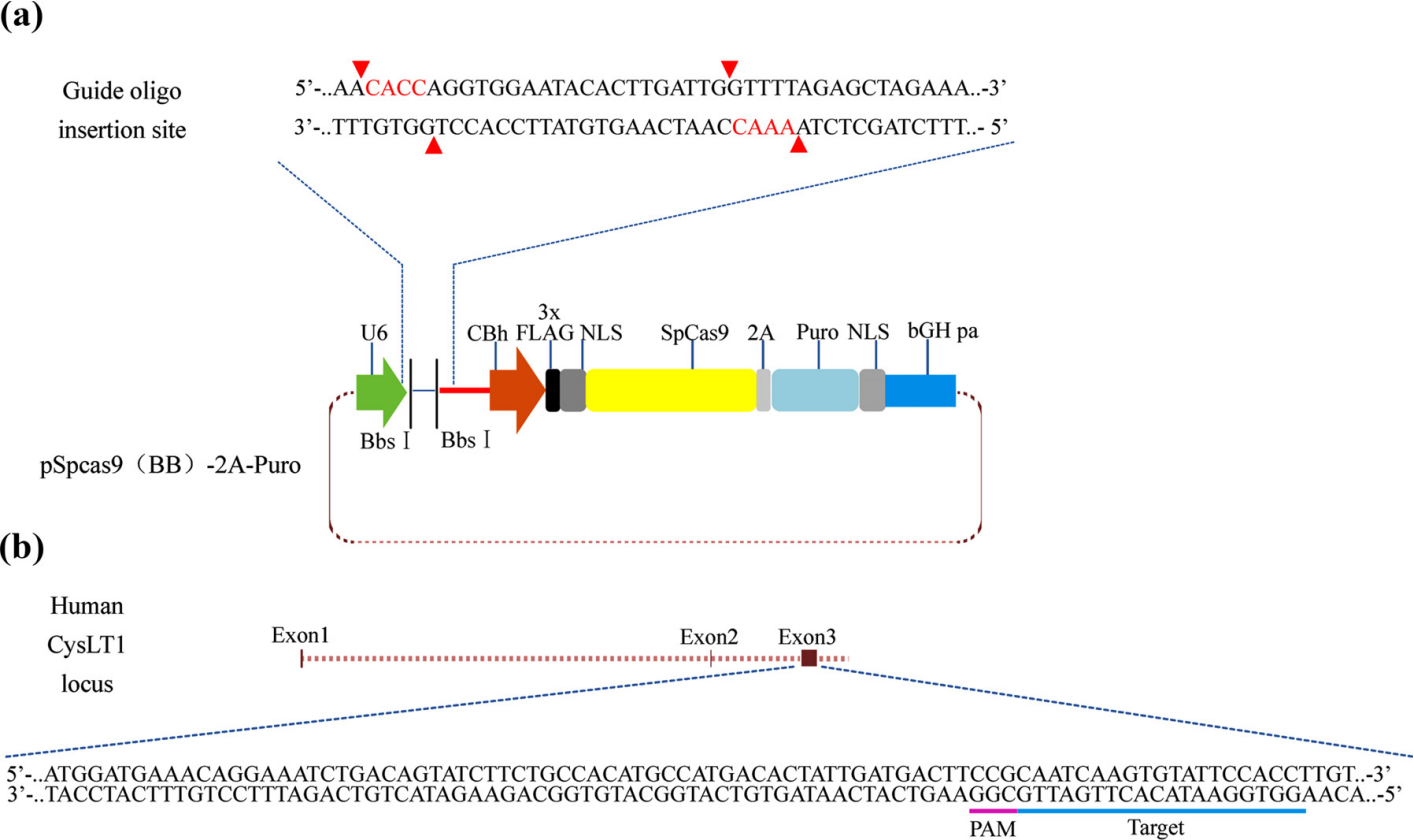
Schematic diagram of PX459 plasmid and target site of sgRNA. (**a**) Red triangle indicates the cleavage site of BbsI, which was used for sgRNA insertion. (**b**) Protospacer adjacent motif (PAM) and sgRNA recognition site (target) in exon 3.

**Fig. (6):**
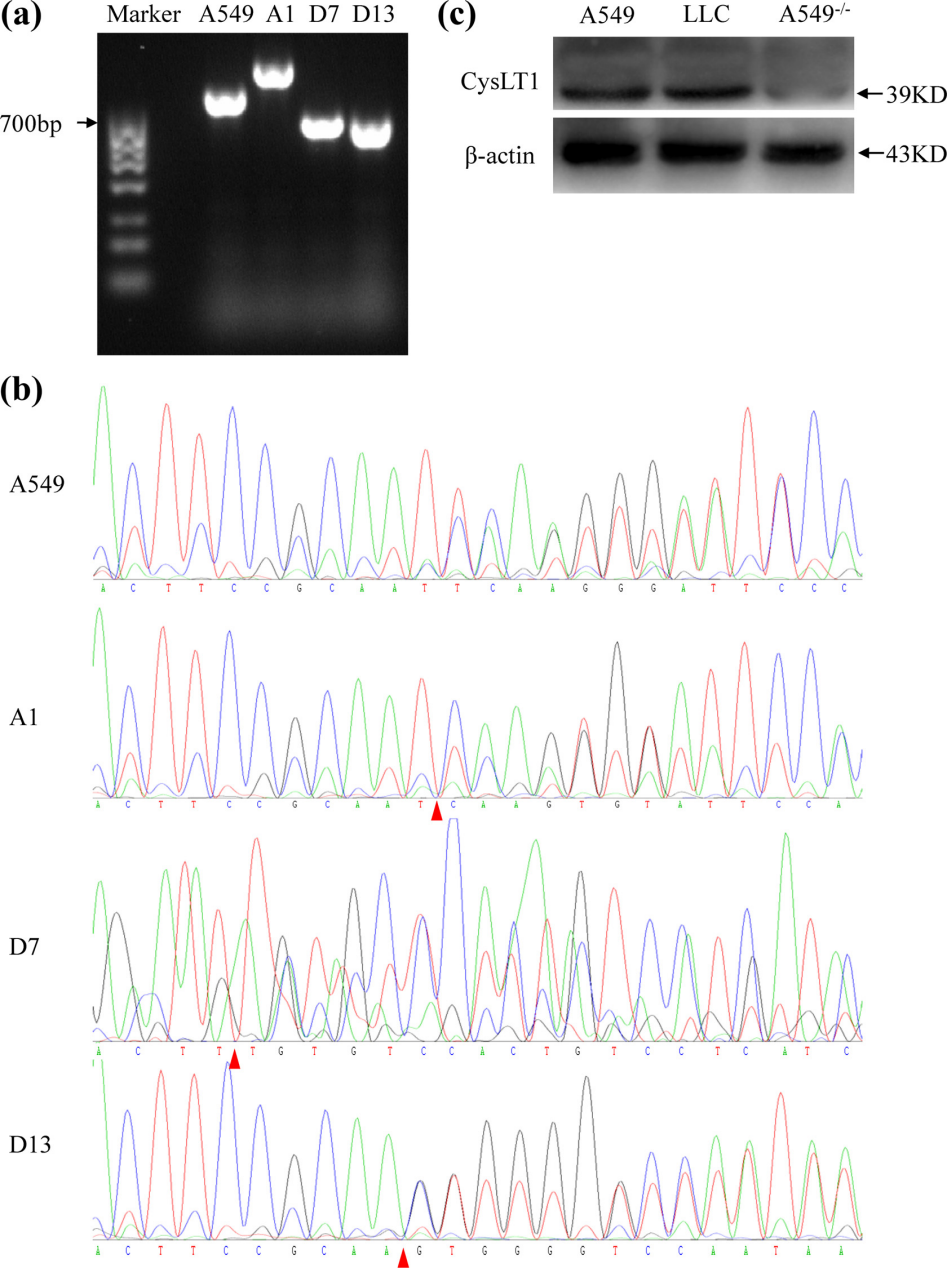
Identification of *CysLT1*-knockout cell lines. (**a**) Results of gel electrophoresis. (**b**) Results of Sanger sequencing. Red triangles indicate mutation sites. (**c**) Results of western blot analysis.

To validate the mechanisms by which montelukast blocked cell migration, we constructed a *CysLT1*-knockout cell line. To establish *CysLT1*-knockout clones, we constructed a PX459 plasmid containing sgRNA targeting exon 3 of human *CysLT1* (Figs. **5a** and **b**). Based on the results of gel electrophoresis (Fig. **6a**), we selected three *CysLT1*-knockout A549 cell lines (A1, D7, and D13) with large fragment insertions/deletions. *CysLT1* was recognized by sgRNA, and several base pairs were knocked out by Cas9. Simultaneously, non-homologous end-joining repair was conducted at this site. Sanger sequencing of the PCR products confirmed that a 144-bp fragment was inserted into exon 3 of *CysLT1* in the A1 cell line, a 170-bp sequence was deleted from the D7 cell line, and a 190-bp region was absent from the D13 cell line. Cas9 recognized specific sites in *CysLT1* in all three cell lines. A frameshift mutation occurred in the exon-coding region of *CysLT1* (Fig. **6b**). The D7 cell line was used in subsequent experiments (hereafter designated A549^−/−^). To verify the absence of CysLT1 expression in A549^−/−^ cells, total protein was collected, and western blotting was performed (Fig. **6c**) using A549 and LLC cells as controls. The results confirmed that *CysLT1* was knocked out in A549^−/−^ cells.

### 3.5 *CysLT1* Knockout Inhibits A549 Cell Migration

**Fig. (7):**
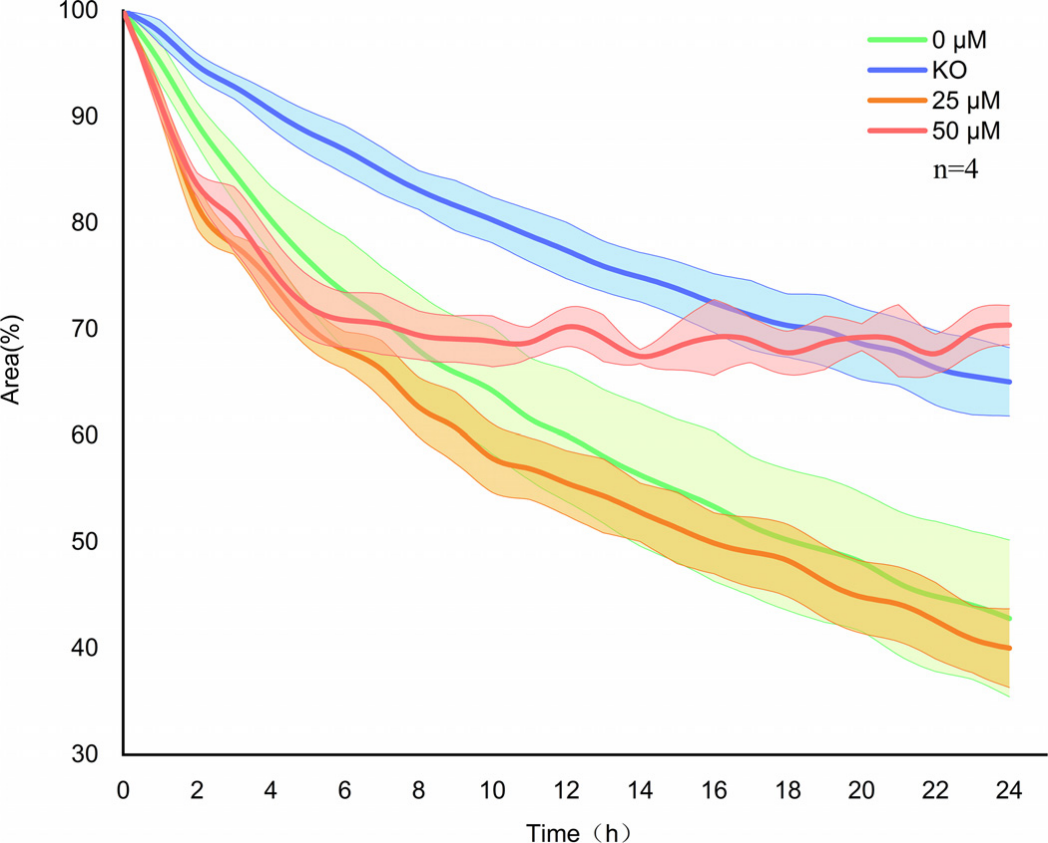
*CysLT1* knockout inhibits migration of A549 cells. Migration ability was detected in the presence or absence of different montelukast concentrations after 24 h using wound-healing assays. The ratio of the wound-healing area was calculated using high-content imaging. 0 µM: untreated A549 cells; KO: *CysLT1*-knockout A549^−/−^ cells; 25 µM: A549 cells treated with 25 µM montelukast; 50 µM: A549 cells treated with 50 µM montelukast. Data are presented as means±SDs (n = 4). *p < 0.05.

To explore the influence of CysLT1 on the migration of lung cancer cells, cell migration ability was detected in wound-healing assays using A549^−/−^ cells and wild-type A549 cells treated with different concentrations of montelukast. Knockout of *CysLT1* inhibited the A549^−/−^ cell migration ability; these cells showed considerably slower migration than control cells (Fig. **7**). Furthermore, 50 μM montelukast noticeably blocked the migration of wild-type A549 cells, whereas low-concentration montelukast had little effect on lung cancer cell migration. These findings confirm that *CysLT1* plays a major role in lung cancer cell migration, and the knockout of the gene effectively inhibited this ability *in vitro.*

## 4 DISCUSSION

We evaluated the molecular mechanisms through which montelukast inhibited cell migration. Our findings show that montelukast decreased A549 cell migration by inhibiting CysLT1 expression. Treatment of lung cancer is limited by a lack of appropriate drugs. However, drug repositioning may be applicable to lung cancer therapy. Compared with new drug research and development, drugs that have already been approved for clinical use are less costly to develop and often show better safety profiles. Tumor development is closely related to chronic inflammation, and inflammation is a hallmark of lung cancer tumorigenesis; particularly, pro-inflammatory chemokines and cytokines are present in the tumor microenvironment [21]. Furthermore, chronic inflammation may explain the high CysLT1 expression in lung cancer cells. Montelukast is an existing drug that can inhibit the binding of LTD4 to the CysLT1 receptor and reduce lung cancer risk [22]. Previous studies mainly focused on the effects of montelukast on apoptosis induction in lung cancer cells and on metastasis inhibition via decreasing capillary permeability [19, 23]. These studies suggested that montelukast can be applied in lung cancer therapy. Therefore, we evaluated whether montelukast affects lung cancer pathogenesis via CysLT1. CysLT1 is closely related to tumor cell migration. The CysLT1 receptor promotes the migration of lung cancer and colon cancer cells following activation by LTD4 [15, 24]. In addition, patients with prostate cancer and epithelioma exhibit higher CysLT1 expression compared to healthy individuals [25]. Inhibition of cell proliferation and promotion of apoptosis were observed after induction with a CysLT1 antagonist in prostate cancer cells [26]. Importantly, we found that low-concentration montelukast (25 μM) had little effect on CysLT1 expression in A549 cells, explaining why this concentration of montelukast did not affect lung cancer cell migration. However, cell migration decreased when CysLT1 was inhibited using a higher concentration of montelukast (50 μM). In contrast, murine LLC cells were more sensitive to montelukast and showed responses even at lower concentrations. This discrepancy may be related to differences in the subcellular localization of CysLT1 among cell lines. As a G-protein-coupled receptor, CysLT1 must localize to the cell membrane to exert its biological functions. Because more CysLT1 was localized on the cell membrane in LLC cells, migration inhibition by montelukast was stronger in LLC cells. Next, we examined the effects of CysLT1 on migration using a *CysLT1*-knockout cell line (A549^−/−^ cells). Our results demonstrated that decreased CysLT1 expression following montelukast treatment was essential for the inhibition of cell migration. A major limitation of currently available treatment strategies is related to the pattern of cell migration; indeed, several therapies targeting specific cell patterns cannot completely prevent tumor metastasis [27], and multiple mechanisms of cell migration may be involved. Further work is required to clarify this possibility.

## CONCLUSION

As a highly expressed receptor, CysLT1 shows potential as a therapeutic target for inhibiting lung cancer metastasis, and montelukast may be useful as adjuvant therapy in lung cancer treatment.

## Data Availability

Data sharing is not applicable to this article as no datasets were generated or analyzed during the current study.

## LIST OF ABBREVIATIONS

CysLT1: Cysteinyl Leukotriene Receptor 1
FBS: Fetal Bovine Serum
NSCLC: Non-small Cell Lung Cancer
sgRNA: Single Guide RNA

## References

[1] Gao S., Li N., Wang S., Zhang F., Wei W., Li N., Bi N., Wang Z., He J. (2020). Lung cancer in people’s Republic of China.. J. Thorac. Oncol..

[2] Hosonaga M., Saya H., Arima Y. (2020). Molecular and cellular mechanisms underlying brain metastasis of breast cancer.. Cancer Metastasis Rev..

[3] Boire A., Brastianos P.K., Garzia L., Valiente M. (2020). Brain metastasis.. Nat. Rev. Cancer.

[4] Yousefi M., Bahrami T., Salmaninejad A., Nosrati R., Ghaffari P., Ghaffari S.H. (2017). Lung cancer-associated brain metastasis: Molecular mechanisms and therapeutic options.. Cell. Oncol..

[5] Page S., Milner-Watts C., Perna M., Janzic U., Vidal N., Kaudeer N., Ahmed M., McDonald F., Locke I., Minchom A., Bhosle J., Welsh L., O’Brien M. (2020). Systemic treatment of brain metastases in non-small cell lung cancer.. Eur. J. Cancer.

[6] Du W., Zhu J., Zeng Y., Liu T., Zhang Y., Cai T., Fu Y., Zhang W., Zhang R., Liu Z., Huang J. (2021). KPNB1-mediated nuclear transloca-tion of PD-L1 promotes non-small cell lung cancer cell proliferation via the Gas6/MerTK signaling pathway.. Cell Death Differ..

[7] Ramalingam S.S., Vansteenkiste J., Planchard D., Cho B.C., Gray J.E., Ohe Y., Zhou C., Reungwetwattana T., Cheng Y., Che-waskulyong B., Shah R., Cobo M., Lee K.H., Cheema P., Tiseo M., John T., Lin M.C., Imamura F., Kurata T., Todd A., Hodge R., Saggese M., Rukazenkov Y., Soria J.C. (2020). Overall survival with osimertinib in untreated, EGFR-mutated advanced NSCLC.. N. Engl. J. Med..

[8] Todoric J., Antonucci L., Karin M. (2016). Targeting inflammation in cancer prevention and therapy.. Cancer Prev. Res..

[9] Cavanagh M.M., Weyand C.M., Goronzy J.J. (2012). Chronic inflammation and aging: DNA damage tips the balance.. Curr. Opin. Immunol..

[10] Kawanishi S., Ohnishi S., Ma N., Hiraku Y., Murata M. (2017). Crosstalk between DNA damage and inflammation in the multiple steps of carcinogenesis.. Int. J. Mol. Sci..

[11] Gomes M., Teixeira A.L., Coelho A., Araújo A., Medeiros R. (2014). The role of inflammation in lung cancer.. Adv. Exp. Med. Biol..

[12] Horn V., Triantafyllopoulou A. (2018). DNA damage signaling and polyploid macrophages in chronic inflammation.. Curr. Opin. Immunol..

[13] Tian W., Jiang X., Kim D., Guan T., Nicolls M.R., Rockson S.G. (2020). Leukotrienes in tumor-associated inflammation.. Front. Pharmacol..

[14] Yamamoto T., Miyata J., Arita M., Fukunaga K., Kawana A. (2019). Current state and future prospect of the therapeutic strategy targeting cys-teinyl leukotriene metabolism in asthma.. Respir. Investig..

[15] Lukic A., Wahlund C.J.E., Gómez C., Brodin D., Samuelsson B., Wheelock C.E., Gabrielsson S., Rådmark O. (2019). Exosomes and cells from lung cancer pleural exudates transform LTC4 to LTD4, promoting cell migration and survival via CysLT1.. Cancer Lett..

[16] Yokomizo T., Nakamura M., Shimizu T. (2018). Leukotriene receptors as potential therapeutic targets.. J. Clin. Invest..

[17] Wendell S.G., Fan H., Zhang C. (2020). G protein-coupled receptors in asthma therapy: Pharmacology and drug action.. Pharmacol. Rev..

[18] Piromkraipak P., Sangpairoj K., Tirakotai W., Chaithirayanon K., Unchern S., Supavilai P., Power C., Vivithanaporn P. (2018). Cysteinyl leukotriene receptor antagonists inhibit migration, invasion, and expression of MMP-2/9 in human glioblastoma.. Cell. Mol. Neurobiol..

[19] Tsai M.J., Chang W.A., Tsai P.H., Wu C.Y., Ho Y.W., Yen M.C., Lin Y.S., Kuo P.L., Hsu Y.L. (2017). Montelukast induces apoptosis-inducing factor-mediated cell death of lung cancer cells.. Int. J. Mol. Sci..

[20] Zhu L., Maruvada R., Sapirstein A., Peters-Golden M., Kim K.S. (2017). Cysteinyl leukotrienes as novel host factors facilitating Cryptococcus neoformans penetration into the brain.. Cell. Microbiol..

[21] Landskron G., De la Fuente M., Thuwajit P., Thuwajit C., Hermoso M.A. (2014). Chronic inflammation and cytokines in the tumor microenvi-ronment.. J. Immunol. Res..

[22] Tsai M.J., Wu P.H., Sheu C.C., Hsu Y.L., Chang W.A., Hung J.Y., Yang C.J., Yang Y.H., Kuo P.L., Huang M.S. (2016). Cysteinyl leukotri-ene receptor antagonists decrease cancer risk in asthma patients.. Sci. Rep..

[23] Nozaki M., Yoshikawa M., Ishitani K., Kobayashi H., Houkin K., Imai K., Ito Y., Muraki T. (2010). Cysteinyl leukotriene receptor antago-nists inhibit tumor metastasis by inhibiting capillary permeability.. Keio J. Med..

[24] Salim T., Sand-Dejmek J., Sjölander A. (2014). The inflammatory mediator leukotriene D4 induces subcellular β-catenin translocation and mi-gration of colon cancer cells.. Exp. Cell Res..

[25] Matsuyama M., Hayama T., Funao K., Kawahito Y., Sano H., Takemoto Y., Nakatani T., Yoshimura R. (2007). Overexpression of cysteinyl LT1 receptor in prostate cancer and CysLT1R antagonist inhibits prostate cancer cell growth through apoptosis.. Oncol. Rep..

[26] Tang C., Lei H., Zhang J., Liu M., Jin J., Luo H., Xu H., Wu Y. (2018). Montelukast inhibits hypoxia inducible factor-1α translation in pros-tate cancer cells.. Cancer Biol. Ther..

[27] Eddy C.Z., Raposo H., Manchanda A., Wong R., Li F., Sun B. (2021). Morphodynamics facilitate cancer cells to navigate 3D extracellular matrix.. Sci. Rep..

